# The positive association between internal migration and hospitalization among the older adults in China: Regional heterogeneity and chronic disease management

**DOI:** 10.3389/fpubh.2022.977563

**Published:** 2022-09-01

**Authors:** Huixiang Zhong, Jin Yang, Na Zhao, Xu Li, Yanli Zhang

**Affiliations:** ^1^National Institute of Hospital Administration, National Health Commission of the People's Republic of China, Beijing, China; ^2^Department of Medical Record Management Statistics, Affliated Huadu Hospital, Southern Medical University, Guangzhou, Guangdong, China; ^3^Department of Endocrinology and Metabolism, Peking University Third Hospital, Peking University, Beijing, China; ^4^Outpatient Department, Yantai Affliated Hospital of Binzhou Medical University, Yantai, Shandong, China

**Keywords:** older adults, migration, inpatient services, primary care, chronic disease

## Abstract

**Background:**

Post-retirement migrants are rapidly increasing in China, but the impact of internal migration on hospitalization among older adults remains under-researched. Understanding this impact is essential for health policies development and improvement. This study aims to identify the most vulnerable population, evaluate the association between migration and hospitalization, and discuss potential causes of the association.

**Methods:**

14,478 older adults were extracted from the 2018 to 2019 Chinese Longitudinal Healthy Longevity Survey (CLHLS) database and divided into four groups according to migration experience and age at migration: non-migrants, pre-adulthood migrants, pre-retirement migrants, and post-retirement migrants. Post-retirement migrants were key research subjects. We employed Pearson's chi-square test to compare group differences in outcome and covariates, and multivariate logistic regression analysis to examine the association between migration and hospitalization by regions and chronic conditions.

**Results:**

Significant intergroup differences were observed in demographic characteristics, socioeconomic factors, health habits, and health-related factors. Post-retirement migrants displayed following characteristics: female predominance (61.6%; 1,472/2,391), tending toward urban areas (80.9%; 1,935/2,391), and the highest prevalence rate of chronic disease (46.7%; 1,116/2,391). Urban migrants in eastern China were more likely to be hospitalized (OR = 1.65; 95% CI: 1.27–2.15), especially those who were diagnosed with chronic disease (OR = 1.51; 95% CI: 1.04–2.19) or with unconfirmed chronic conditions (OR = 1.98; 95% CI: 1.36–2.89).

**Conclusions:**

Internal migration is associated with the hospitalization of post-retirement migrants moving to eastern China. Improved chronic disease management and early interventions might lower the hospitalization. Effective policies should be formulated to reduce the disparity in primary care services across China, thereby facilitating the access of migrants to these services.

## Introduction

Internal migration, the movement within countries, has become a common occurrence in China. Motivated by caring for the younger generation, elderly care or reunion with family and jobs (1), older adults out of the migrant population have been increasing. According to the report on China's migrant population development 2015 issued by the National Health Commission of the People's Republic of China (2), the older migrants had accounted for 5.3 percent (13,040,000/247,000,000) of the total migrants. Given the older migrants' annual growth rate (6.6 percent) and the extended life expectancy since 2016 (3, 4), older migrants are expected to rise steadily in China.

Older adults' redistribution in space necessitates the redistribution of health resource (5). Since primary care and health insurance in China are intimately tied to the household registration system (6, 7), migration unavoidably decreases the migrants' access to health services (8, 9). Thus far the declining access has been demonstrated in many aspects except inpatient services. However, hospitalization provides both vital evidence for the effectiveness of primary care services and reliable indication of the impact of migration on migrants' health (10, 11). As such, clarifying the association between migration and hospitalization is critical for identifying at-risk groups, and then enabling policy makers to develop or improve subsequent healthcare policy.

Elderly population over 65 years old in China has reached 191 million in 2020 (12), three-quarters of whom has at least one chronic disease (13). Aiming to provide the same basic public health services (BPHS) to all citizens, the Chinese government has been implementing BPHS equalization program from 2009 onwards (14). By BPHS, every Chinese older adult should have had access to free health management services, and chronic disease health management for those who suffer from hypertension and diabetes mellitus. Nonetheless, older migrants' utilization of BPHS has been found insufficient (15–17). Considerable migrants receive deficient chronic disease management (18). In addition, previous research has discovered a strong substitution element between outpatient services and inpatient services (19). Owing to the non-portability of health insurance (20), Chinese migrants are unable to meet the expenses at the location where they receive outpatient services, thereby reducing outpatient visits and preferring treatment delay (21, 22). Inadequate access to care has been linked to physical disability, cognitive impairment, and an increased risk of mortality (23).

In light of the above, it is reasonable to conjecture that older migrants would use more inpatient services in the context of the limited effectiveness of primary care and the reduction in outpatient care. As yet this assumption has not been confirmed in China, and to some extent this will be tested in this study.

China exhibits an uneven distribution of medical resources and a wide variation in medical standards between urban and rural areas, as well as across provinces (24, 25). In this regard, the association between migration and hospitalization is presumed to be significant in certain areas. To identify these areas will allow for more targeted interventions.

This study describes the characteristics of post-retirement migrants and evaluates the association between migration and hospitalization by comparing them with non-migrants. Considering the regional heterogeneity of migrants, the association would be evaluated stratified by urban-rural distribution and geographical region, and then further stratified by chronic disease so as to study the effectiveness of health management for migrants with chronic conditions.

## Materials and methods

### Data source and participants

Data were extracted from the Eighth Chinese Longitudinal Healthy Longevity Survey (CLHLS) conducted between 2018 and 2019, which extended its scope of the investigation into 23 mainland provinces to represent approximately 85% of the Chinese elderly population (≥65 years) (26). The CLHLS adopted several measures to provide comparative, accurate, and representative information, including the development of internationally accepted questionnaires, face-to-face interviews, and double data entry (27). All CLHLS data are available at Peking University Open Research Data Platform.

**Figure 1:**
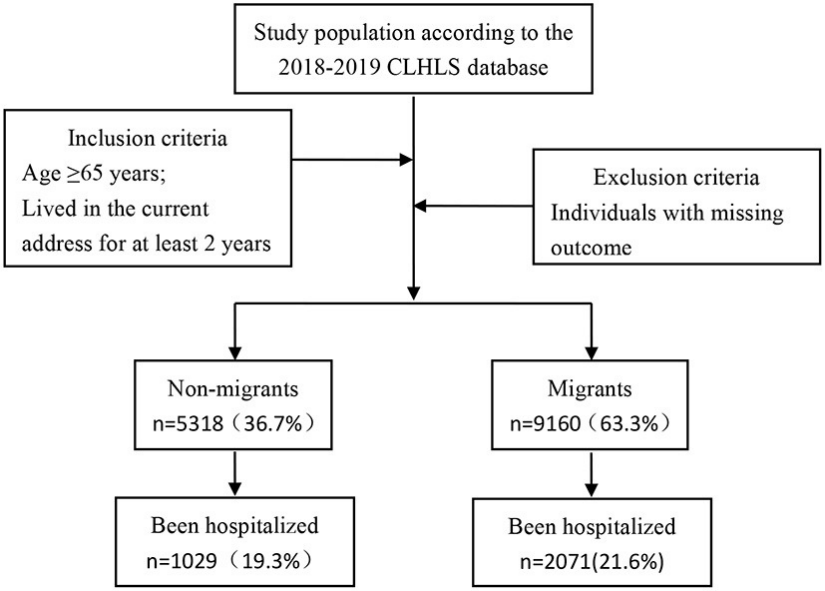
Flowchart of sample selection based on 2018–2019 CLHLS data.

A total of 15,874 individuals aged 65 years or older, having lived in their current address for at least 2 years, were selected from the 2018 CLHLS database (Figure 1). Individuals with missing outcome were excluded. For some variables with missing data, we performed multiple imputation instead of deleting (28). A total of 14,478 participants were included in the analysis.

### Migration

Internal migration could be defined by the respondents' age and the following questions: (1) “How many years did you live in the current address?” and (2) “Do you currently live in the city you were born?”. Migrants were defined as the respondents who answered “more than 2 years” to the first question and “no” to the second question; and non-migrants were defined as the respondents who answered the years same as age to the first question and “yes” to the second question. For the convenience of expression, internal migration is denoted as migration in the following paragraphs.

Age at migration was the core explanatory variables.

As several research have highlighted the importance of seeing migration within a life course context with certain life course events such as employment or retirement (29), we divided age at migration into three categories: pre-adulthood (1–17 years), pre-retirement (18–59 years for male, 18–54 years for female), and post-retirement (60 years and above for male, 55 years and above for female). The post-retirement migrants would be focused on in this study. By directly comparing with non-migrants and indirectly comparing with pre-adulthood and pre-retirement migrants, more characteristics of post-retirement migrants which associate with hospitalization are expected to be discovered.

Migrants whose destination located in urban areas were referred to as urban migrants, while others were rural migrants.

### Outcome variable

The outcome variable was hospitalization. A questionnaire was applied to determine the number of patients hospitalized in the past 2 years using a dichotomous response, in which “yes” corresponded to “hospitalized at least once” and “no” corresponded to “not hospitalized.”

### Covariates

As shown in Table 1, Demographic variables included gender (male, female), age (65–79, 80–94, ≥95 years), education attainment (never educated, primary school, middle school, college and above), residential area (urban, rural), and geographical region (north, northeast, east, central-south, and west). The classification of geographical region was based on CLHLS guidelines. The study cohort lived in the following cities: Tianjin, Hebei, Shanxi, and Beijing (north), Liaoning, Jilin, and Heilongjiang (northeast), Jiangsu, Zhejiang, Anhui, Fujian, Jiangxi, and Shanghai (east), Hainan, Henan, Hunan, Hubei, Guangdong, and Guangxi (central-south), Sichuan, Shanxi, and Chongqing (west).

**Table 1:** Profile of the study cohort according to covariates.

**Demographic variables**	**Socioeconomic factors**	**Health habits**	**Health-related factors**
1. Gender	1. Former occupation	1. Smoking	1. Health status
2. Age	2. Monthly pension	2. Alcohol use	2. Change in health status
3. Education attainment	3. Financial supporter	3. Exercise	3. Chronic disease
4. Residential area	4. Health care payer	4. Physical examination	4. Loneliness
5. Geographical region			5. Anxiety

Socioeconomic variables included former occupation (agriculture, professional and managerial, others), monthly pension (including retirement and insurance pension, measured in RMB; 0, 0–2,000, >2,000), financial supporter (owner, family, others), and health care payer (insurance company, household member, or others). Former occupation came from the question “what is your occupation before the age of 60 years?”. For ease of comparison, several options were combined, and agriculture, forestry, animal husbandry, and fishery workers were included in the “agriculture” category, professional and technical personnel and governmental, institutional, and managerial personnel were included in the “professional and managerial” category, whereas other occupations were assigned to the “others” category. Similar combination occurred in the topic “health care payer,” and “owner” remains “owner,” “urban employee/resident medical insurance,” “cooperative” and “private” were aggregated into “insurance,” “spouse” and “children” were aggregated into “household members,” and other payers were aggregated into “others.”

Health habits were smoking (yes, no), alcohol use (yes, no), exercise (yes, no), and physical examination (yes, no).

Health-related factors were health status (good, average, bad), change in health status (better, no change, worse), chronic disease (yes, unconfirmed, no), loneliness (yes, no) and anxiety (yes, no). Both health status and change in health status were self-reported, and derived from the questions “how do you rate your health at present?” and “how do you rate your health at present compared with one year ago?”, respectively.

### Statistical analysis

Group differences in outcome and covariates were compared using the Pearson's chi-square test. The association between age at migration, covariates and hospitalization was assessed by univariate logistic regression analysis before constructing multivariate regression models. Multivariate binary logistic regression analysis with LR forward-stepwise method, of which put in criteria is *p* < 0.05 and removed in *p* > 0.10, were performed to determine the variables significantly associated with hospitalization. The LR forward-stepwise method guarantees the absence of multicollinearity between variables entering the models. The reliability of the multivariate regression model was analyzed using the Hosmer and Lemeshow goodness-of-fit test (30), and the goodness-of-fit was considered acceptable if the value of the chi-square statistic was low accompanied by *p* > 0.05 in this test. Statistical significance in this study was defined at a *p*-value which <0.05. Statistical analysis was conducted using SPSS version 25.0 (IBM, SPSS Inc, Chicago, IL, USA).

## Results

### Descriptive statistics

#### Sample characteristics

Table 2 summarizes sample characteristics by age at migration. 25.6% of the post-retirement migrants had been hospitalized within 2 years, and the proportion was significantly higher than other subgroups.

**Table 2:** Profile of the study cohort.

**Variable**	**Item**	**Total cohort (*n*, %)**	**Non-migrants (*n*, %)**	**Age at migration (** ***n*** **, %)**			**χ^2^**	***p*-Value**
				**Pre-adulthood**	**Pre-retirement**	**Post-retirement**		
Total		14,478 (100.0)	5,318 (36.7)	1,757 (12.1)	5,012 (34.6)	2,391 (16.5)		
Gender	Male	6,385 (44.1)	3,640 (68.4)	575 (32.7)	1,251 (25.0)	919 (38.4)	2,146.841	<0.001
	Female	8,093 (55.9)	1,678 (31.6)	1,182 (67.3)	3,761 (75.0)	1,472 (61.6)		
Age	65–79	4,969 (34.3)	2,126 (40.0)	493 (28.1)	1,877 (37.5)	473 (19.8)	477.287	<0.001
	80–94	5,663 (39.1)	2,099 (39.5)	762 (43.4)	1,830 (36.5)	972 (40.7)		
	≥95	3,846 (26.6)	1,093 (20.6)	502 (28.6)	1,305 (26.0)	946 (39.6)		
Education attainment	Never educated	7,012 (48.4)	2,288 (43.0)	1,055 (60.0)	2,606 (52.0)	1,063 (44.5)	434.468	<0.001
	Primary school	5,024 (34.7)	2,232 (42.0)	530 (30.2)	1,477 (29.5)	785 (32.8)		
	Middle school	2,053 (14.2)	737 (13.9)	143 (8.1)	763 (15.2)	410 (17.1)		
	College and above	389 (2.7)	61 (1.1)	29 (1.7)	166 (3.3)	133 (5.6)		
Residential area	Urban	7,950 (54.9)	2,392 (45.0)	815 (46.4)	2,808 (56.0)	1,935 (80.9)	919.660	<0.001
	Rural	6,528 (45.1)	2.926 (55.0)	942 (53.6)	2,204 (44.0)	456 (19.1)		
Geographical region	North	839 (5.8)	151 (2.8)	59 (3.4)	339 (6.8)	290 (12.1)	753.696	<0.001
	Northeast	605 (4.2)	87 (1.6)	51 (2.9)	276 (5.5)	191 (8.0)		
	East	5,922 (40.9)	2,443 (45.9)	669 (38.1)	2,040 (40.7)	770 (32.2)		
	Center-South	5,246 (36.2)	2,055 (38.6)	738 (42.0)	1,799 (35.9)	654 (27.4)		
	West	1,866 (12.9)	582 (10.9)	240 (13.7)	558 (11.1)	486 (20.3)		
Former occupation	Agriculture	7,704 (53.2)	3,360 (63.2)	1,009 (57.4)	2,539 (50.7)	796 (33.3)	889.303	<0.001
	Professional and managerial	3,441 (23.8)	837 (15.7)	260 (14.8)	1,328 (26.5)	1,016 (42.5)		
	Others	3,333 (23.0)	1,121 (21.1)	488 (27.8)	1,145 (22.8)	579 (24.2)		
Monthly pension (RMB)	0	7,047 (48.7)	2,903 (54.6)	966 (55.0)	2,324 (46.4)	854 (35.7)	958.234	<0.001
	1–2,000	4,355 (30.1)	1,811 (34.1)	561 (31.9)	1,410 (28.1)	573 (24.0)		
	>2,000	3,076 (21.2)	604 (11.4)	230 (13.1)	1,278 (25.5)	964 (40.3)		
Financial supporter	Owner	4,699 (32.5)	1,577 (29.7)	392 (22.3)	1,697 (33.9)	1,033 (43.2)	276.337	<0.001
	Family	7,205 (49.8)	2,767 (52.0)	1,056 (60.1)	2,480 (49.5)	902 (37.7)		
	Others	2,574 (17.8)	974 (18.3)	309 (17.6)	835 (16.7)	456 (19.1)		
Health care payer	Owner	1,851 (12.8)	792 (14.9)	179 (10.2)	603 (12.0)	277 (11.6)	70.506	<0.001
	Medical insurance	7,885 (54.5)	2,787 (52.4)	948 (54.0)	2,739 (54.6)	1,411 (59.0)		
	Family	4,030 (27.8)	1,474 (27.7)	554 (31.5)	1,428 (28.5)	574 (24.0)		
	Others	712 (4.9)	265 (5.0)	76 (4.3)	242 (4.8)	129 (5.4)		
Smoking	Yes	2,171 (15.0)	1,238 (23.3)	219 (12.5)	450 (9.0)	264 (11.0)	466.82	<0.001
	No	12,307 (85.0)	4,080 (76.7)	1,538 (87.5)	4,562 (91.0)	2,127 (89)		
Alcohol use	Yes	2,074 (14.3)	1,048 (19.7)	230 (13.1)	531 (10.6)	265 (11.1)	204.98	<0.001
	No	12,404 (85.7)	4,270 (80.3)	1,527 (86.9)	4,481 (89.4)	2,126 (88.9)		
Exercise	Yes	4,318 (29.8)	1,499 (28.2)	454 (25.8)	1,559 (31.1)	806 (33.7)	41.315	<0.001
	No	10,160 (70.2)	3,819 (71.8)	1,303 (74.2)	3,453 (68.9)	1,585 (66.3)		
Physical examination	Yes	9,807 (67.7)	3,870 (72.8)	1,172 (66.7)	3,425 (68.3)	1,340 (56.0)	212.966	<0.001
	No	4,671 (32.3)	1,448 (27.2)	585 (33.3)	1,587 (31.7)	1,051 (44.0)		
Health status	Good	6,266 (43.3)	2,407 (45.3)	688 (39.2)	2,133 (42.6)	1,038 (43.4)	29.188	<0.001
	Average	6,333 (43.7)	2,240 (42.1)	793 (45.1)	2,244 (44.8)	1,056 (44.2)		
	Bad	1,879 (13.0)	671 (12.6)	276 (15.7)	635 (12.7)	297 (12.4)		
Change in health status	Better	1,733 (12.0)	604 (11.4)	182 (10.4)	644 (12.8)	303 (12.7)	19.531	0.003
	no change	7,934 (54.8)	3,006 (56.5)	949 (54.0)	2,698 (53.8)	1,281 (53.6)		
	Worse	4,811 (33.2)	1,708 (32.1)	626 (35.6)	1,670 (33.3)	807 (33.8)		
Chronic disease	Yes	5,999 (41.4)	2,123 (39.9)	692 (39.4)	2,068 (41.3)	1,116 (46.7)	70.217	<0.001
	Unconfirmed	5,754 (39.7)	2,060 (38.7)	706 (40.2)	2,079 (41.5)	909 (38.0)		
	No	2,725 (18.8)	1,135 (21.3)	359 (20.4)	865 (17.3)	366 (15.3)		
Loneliness	Yes	5,071 (35.0)	1,745 (32.8)	647 (36.8)	1,768 (35.3)	911 (38.1)	24.012	<0.001
	No	9,407 (65.0)	3,573 (67.2)	1,110 (63.2)	3.244 (64.7)	1,480 (61.9)		
Anxiety	Yes	4,547 (31.4)	1,481 (27.8)	611 (34.8)	1,638 (32.7)	817 (34.2)	52.758	<0.001
	No	9.931 (68.6)	3,837 (72.2)	1,146 (65.2)	3,374 (67.3)	1,574 (65.8)		
Hospitalization	Yes	3,100 (21.4)	1,029 (19.3)	387 (22.0)	1,073 (21.4)	611 (25.6)	38.219	<0.001
	No	11,378 (78.6)	4,289 (80.7)	1,370 (78.0)	3,939 (78.6)	1,780 (74.4)		

A total of 55.9% of the respondents were females, and the male-to-female ratios of migrants and that of non-migrants were completely contrary. 2,391 older adults migrated to their current residence after retirement, accounting for 16.5% of total participants, and 80.9% (1,351/2,391) of them migrated across urban areas; in contrast, migration before adulthood or retirement were more likely to happen in rural areas, 52.4% (920/1,750) and 42.9% (2,150/5,012), respectively. Migrants who migrated after retirement showed two characteristics compared with other subsets, the lowest illiteracy rate (44.5%; 1,063/2,391) and the most dispersed geographical distribution.

With regard to socioeconomic status, agricultural practitioners dominated a prime position, except for those who migrated after retirement. 48.7% of the respondents did not receive pensions; accordingly, 49.7% relied on family for living expenses. 54.5% had health insurance paid by the insurance company. An upward trend in socioeconomic status was clear as the age of migration increased.

Comparing health habits revealed that those who migrated later were more likely to develop positive health habits.

43.7% of the participants reported a good health status while 13.0% reported bad, 33.2% deemed their health status worsened, and 63.3% were diagnosed with chronic disease. Loneliness and anxiety were more commonly reported by post-retirement migrants. It's noteworthy that the highest health satisfaction degree (43.4%; 1,038/2,391), prevalence rate of chronic disease (46.7%; 1,116/2,391) occurred simultaneously in the post-retirement migration group.

There were significant intergroup differences in migration-related variables, demographic variables, socioeconomic factors, health habits, health-related factors and outcome variable.

### Regression analyses

#### Effect of age at migration and covariates on hospitalization

From Table 3, it could be found that age at migration and all the covariates were positively associated with hospitalization. The effect of age at migration on hospitalization slightly decreased when adjusting for all the covariates [Model 1; Hosmer and Lemeshow goodness-of-fit χ^2^ (8df) =5.813, *p* = 0.668], still, older adults who migrated after retirement remained the most likely to be hospitalized. As the positive association occurred in urban area [Model 2; Hosmer and Lemeshow goodness-of-fit χ^2^ (8df) =4.655, *p* = 0.794] rather than in rural area [Model 3; Hosmer and Lemeshow goodness-of-fit χ^2^ (8df) =5.270, *p* = 0.728], participants settled in rural area would be excluded in the following hierarchical analyses.

**Table 3:** Results of logistic regressions on hospitalization in the study cohort.

**Variable**	**Item**	**Univariate analysis**	**Multivariate analysis OR (95% CI)**		
		**OR (95% CI)**	**Model 1**	**Model 2**	**Model 3**
Age at migration (ref: non-migrants)	Pre-adulthood	1.18 (1.03, 1.34)^*^	1.19 (1.04, 1.38)^*^	1.25 (1.02, 1.53)^*^	
	Pre-retirement	1.14 (1.03, 1.25)^**^	1.16 (1.04, 1.30)^**^	1.17 (1.01, 1.36)^*^	
	Post-retirement	1.43 (1.28, 1.60)^***^	1.31 (1.15, 1.50)^***^	1.33 (1.13, 1.57)^***^	
Gender (ref: female)	Male	1.21 (1.12, 1.31)^***^	1.41 (1.27, 1.55)^***^	1.39 (1.23, 1.58)^***^	1.27 (1.10, 1.47)^***^
Age (ref:65–79)	80–94	1.19 (1.09, 1.31)^***^	1.12 (1.01, 1.24)^*^	1.19 (1.04, 1.37)^*^	1.06 (0.91, 1.23)
	≥95	0.83 (0.75, 0.93)^***^	0.92 (0.81, 1.05)	1.09 (0.92, 1.28)	0.75 (0.62, 0.90)^*^
Education attainment (ref: never educated)	Primary school	1.14 (1.04, 1.24)^***^	0.97 (0.88, 1.08)	1.04 (0.90, 1.19)	
	Middle school	1.12 (1.00, 1.26)	0.80 (0.68, 0.92)^**^	0.80 (0.67, 0.97)^*^	
	College and above	1.29 (1.02, 1.63)^*^	0.69 (0.53, 0.90)^**^	0.74 (0.56, 0.99)^*^	
Geographical region (ref: west)	North	0.82 (0.68, 0.99)^*^	0.71 (0.58, 0.88)^**^	0.63 (0.50, 0.80)^***^	
	Northeast	0.80 (0.64, 1.00)^*^	0.80 (0.63, 1.01)	0.75 (0.57, 0.99)^*^	
	East	0.75 (0.67, 0.85)^***^	0.83 (0.73, 0.94)^**^	0.72 (0.61, 0.84)^***^	
	Center-South	0.75 (0.66, 0.85)^***^	0.85 (0.74, 0.97)^*^	0.78 (0.66, 0.92)^**^	
Former occupation (ref: agriculture)	Professional and managerial	1.46 (1.33, 1.61)^***^			
	Others	1.13 (1.02, 1.25)^*^			
Monthly pension (ref:0)	1–2,000	1.32 (1.20, 1.45)^***^	1.25 (1.13, 1.38)^***^	1.15 (0.99, 1.34)	1.35 (1.18, 1.55)^***^
	>2,000	1.75 (1.59, 1.94)^***^	1.61 (1.38, 1.88)^***^	1.51 (1.24, 1.84)^***^	1.73 (1.30, 2.31)^***^
Financial supporter (ref: owner)	Family	0.76 (0.69, 0.83)^***^	1.01 (0.88, 1.16)	0.94 (0.78, 1.13)	
	Others	1.04 (0.93, 1.16)	1.21 (1.05, 1.39)^**^	1.25 (1.05, 1.49)^*^	
Health care payer (ref: owner)	Medical insurance	1.46 (1.28, 1.66)^***^	1.53 (1.33, 1.76)^***^	1.41 (1.18, 1.70)^***^	1.76 (1.42, 2.17)^***^
	Family	1.25 (1.08, 1.44)^***^	1.52 (1.30, 1.78)^***^	1.34 (1.08, 1.67)^**^	1.79 (1.43, 2.25)^***^
	Other	0.79 (0.62, 1.01)	0.92 (0.71, 1.19)	1.04 (0.75, 1.43)	0.74 (0.48, 1.14)
Smoking (ref: no)	Yes	0.85 (0.76, 0.95)^***^			0.81 (0.66, 0.99)^*^
Alcohol use (ref: no)	Yes	0.71 (0.63, 0.80)^***^	0.74 (0.65, 0.85)^***^	0.78 (0.65, 0.93)^;**^	0.74 (0.59, 0.91)^***^
Exercise (ref: no)	Yes	1.18 (1.09, 1.29)^***^	1.11 (1.00, 1.22)^*^		1.33 (1.14, 1.55)^***^
Physical examination (ref: no)	Yes	1.05 (0.97, 1.15)			
Health status (ref: good)	Average	1.55 (1.42, 1.70)^***^	1.41 (1.28, 1.56)^***^	1.44 (1.27, 1.64)^***^	1.39 (1.20, 1.62)^***^
	Bad	3.13 (2.79, 3.52)^***^	2.35 (2.05, 2.7)^***^	2.18 (1.82, 2.61)^***^	2.69 (2.19, 3.29)^***^
Change in health status (ref: better)	No change	0.68 (0.60, 0.78)^***^	0.72 (0.63, 0.82)^***^	0.74 (0.62, 0.88)^***^	0.66 (0.54, 0.82)^***^
	Worse	1.31 (1.15, 1.49)^***^	1.00 (0.87, 1.15)	1.08 (0.90, 1.29)	0.89 (0.71, 1.11)
Chronic disease (ref: no)	Yes	7.81 (6.49, 9.35)^***^	6.45 (5.36, 7.77)^***^	6.73 (5.17, 8.76)^***^	6.37 (4.90, 8.28)^***^
	Unconfirmed	5.41 (4.50, 6.54)^***^	4.48 (3.71, 5.40)^***^	5.08 (3.89, 6.63)^***^	3.94 (3.02, 5.14)^***^
Loneliness (ref: no)	Yes	1.06 (0.98, 1.16)	0.90 (0.82, 0.99)^*^		
Anxiety (ref: no)	Yes	1.35 (1.24, 1.47)^***^	1.16 (1.05, 1.28)	1.13 (1.00, 1.28)^*^	

* p < 0.05.

** p < 0.01.

*** p < 0.01.

OR, odds ratio; 95% CI, 95% confidence interval; ref, reference; Model 1, both urban residents and rural residents were include in the model; Model 2, only urban residents were include; Model 3, only rural residents were included.

In terms of the risk factors of hospitalization, there were obvious urban-rural disparities, as only half of the covariates (gender, age, monthly pension, health care payer, smoking, alcohol use, health status, change in health status, and chronic disease) appeared both in Model 2 and Model 3. Among these common risk factors, chronic disease presented the most noticeable effect.

#### Risk factors of hospitalization by geographical region among urban migrants

With a view to regional economic and health disparities, multivariate logistic regression models with urban residents included were constructed for each geographical region in China. The results in Table 4 show that migration significantly increased the risk of post-retirement migrants' hospitalization in the eastern region only (OR = 1.65; 95% CI: 1.27–2.15).

**Table 4:** Risk factors of hospitalization by geographical region among urban migrants in China.

**Variable**	**Item**	**OR (95% CI)**				
		**North**	**Northeast**	**East**	**Center-South**	**West**
Age at migration (ref: non-migrants)	Pre-adulthood			1.48 (1. 08, 2.04)^*^		
	Pre-retirement			1.13 (0.89, 1.43)		
	Post-retirement			1.65 (1.27, 2.15)^***^		
Gender (ref: female)	Male	1.55 (1.06, 2.26)^*^		1.25 (1. 02, 1.53)^*^		1.46 (1.10, 1.93) ^**^
Age (ref:65–79)	80–94			1.34 (1.08, 1.67)^**^		
	≥95			1.26 (0.97, 1.64)		
Monthly pension (ref:0 RMB)	1–2,000			1.06 (0.83, 1.36)	1.14 (0.90, 1.45)	1.17 (0.84, 1.62)^***^
	>2,000			1.69 (1.23, 2.32) ^***^	1.50 (1.17, 1.92)^**^	1.67 (1.17, 2.40)^***^
Financial supporter (ref: owner)	Family		0.30 (0.14, 0.65)^*^	1.02 (0.75, 1.38)		
	Others		0.37 (0.17, 0.78)^*^	1.62 (1.22, 2.16)^***^		
Health care payer (ref: owner)	Medical insurance		2.06 (0.95, 4.44)			1.87 (1.22, 2.86)^**^
	Family		4.34 (1.56, 12.07)			1.32 (0.78, 2.25)
	Other		1.16 (0.36, 3.73)			1.63 (0.79, 3.38)
Smoking (ref: no)	Yes	0.41 (0.20, 0.84)^*^				
Alcohol use (ref: no)	Yes		0.41 (0.17, 0.98)			0.62 (0.41, 0.93)^*^
Exercise (ref: no)	Yes					
Physical examination (ref: no)	Yes					
Health status (ref: good)	Average			1.65 (1.34, 2.03)^**^	1.69 (1.35, 2.13)^***^	1.38 (1.02, 1.86)^*^
	Bad			2.22 (1.68, 2.94)^***^	2.96 (2.20, 3.98)^***^	2.85 (1.84, 4.42)^***^
Change in health status (ref: better)	No change	0.71 (0.40, 1.26)		0.61 (0.46, 0.81)^***^		0.82 (0.56, 1.20)
	Worse	2.52 (1.41, 4.52)^**^		0.87 (0.64, 1.18)		1.21 (0.80, 1.82)
Chronic disease (ref: no)	Yes	5.55 (2.16, 14.25)^**^	9.74 (2.92, 32.46)^***^	6.63 (4.22, 10.43)^***^	6.45 (4.26, 9.76)^***^	10.27 (4.89, 21.57)^***^
	Unconfirmed	9.51 (3.57, 25.33)^***^	8.38 (2.38, 29.50)^**^	4.66 (2.95, 7.35)^***^	4.44 (2.91, 6.76)^***^	7.44 (3.55, 15.59)^***^
Anxiety (ref: no)	Yes		2.72 (1.61, 4.59) ^***^			

* p < 0.05.

** p < 0.01.

*** p < 0.01.

OR, odds ratio; 95% CI, 95% confidence interval; ref, reference.

#### Impacts of age at migration on hospitalization by chronic disease among urban migrants in Eastern China

For determining whether the risk of hospitalization among eastern China's migrants was associated with chronic conditions, we grouped the urban residents based on their chronic conditions and constructed separate multivariate logistic regression models. As presented in Table 5, after controlling for gender, age, financial supporter, health status, and change in health status, we found that age at migration had no impact on hospitalization by comparing migrants without chronic disease with native counterparts. With respect to migrants diagnosed with chronic disease, pre-adulthood migration and pre-retirement migration were found irrelevant to hospitalization, whereas post-retirement migrants were at a higher risk of hospitalization (OR = 1.51; 95% CI: 1.04–2.19). Notably, the highest OR (1.98; 95% CI: 1.36–2.89) in the study reveals the population group most susceptible to hospitalization, the post-retirement urban migrants with unconfirmed chronic conditions living in eastern China. The above results have strongly suggested that the migrants with chronic conditions might receive inadequate health management.

**Table 5:** OR and 95% CI by chronic disease among urban migrants in eastern China.

**Variable**	**Item**	**Chronic disease**		
		**No**	**Yes**	**Unconfirmed**
Age at migration (ref: non-migrants)	Pre-adulthood		1.39 (0.88, 2.20)	1.58 (0.97, 2.56)
	Pre-retirement		0.90 (0.65, 1.25)	1.56 (1.11, 2.21)^*^
	Post-retirement		1.51 (1.04, 2.19)^*^	1.98 (1.36, 2.89)^***^
Control variables		YES	YES	YES
Constant		9.21	3.92	17.35
Total (n)		770	1,844	1,854

* p < 0.05.

** p < 0.01.

*** p < 0.01.

OR, odds ratio; 95% CI, 95% confidence interval; ref, reference.

## Discussion

This study investigated the distribution of older migrants across China and the association between migration and hospitalization whereby the population most at-risk could be identified. The large sample size increased the reliability of the results. Moreover, the definite chronological order that migration occurred before hospitalization in this study has provided an irreplaceable condition for determining causality further.

### Characteristics of post-retirement migrants

Post-retirement migrants, whether from urban areas or rural areas, were more likely to settle in urban areas. On one hand, the convenient traffic conditions and advanced medical care services in urban areas hold irresistible attraction for older adults in pursuit of better living quality (1). On the other hand, the urbanization process in China has been expanding dramatically, and the urbanization rate was lifted to 63.89% according to the Seventh National Population Census of the People's Republic of China (12).

Females comprised a dominant proportion of migrants. In China, in case of children's migration and resettlement, older women tend to migrate in virtue of strong family ties and a strong sense of responsibility for caring for grandchildren (31).

It seems to be contradictory that post-retirement migrants who reported the highest prevalence rate of chronic disease also declared the best health status. Notwithstanding, the perception of health status varies with each individual and is outside of objective evaluation criteria. The defect of self-assessed health status would be the prime cause of the discrepancy.

### Association between migration and hospitalization

Despite the confounding effect caused by covariates, the risk of hospitalization among all migrants was higher than that among the native population, suggesting the activity of migration did associate with hospitalization.

Prior research has found that early experiences related to migration may have consequences for late-life disease, and the relation is not mitigated by the higher socioeconomic status achieved by early migrants (32). Partially corresponding with this discovery, the risk of being hospitalized among total pre-adulthood migrants ranked higher than that among pre-retirement migrants. Nevertheless, pre-adulthood and pre-retirement migrants with chronic diseases showed insignificant trend toward hospitalization. These two groups of older migrants generally attain indigenous health insurance as natives, hence the better health management in contrast to post-retirement migrants.

Post-retirement migrants who migrated to eastern China was more likely to be hospitalized. Furthermore, the older migrants in unconfirmed chronic conditions displayed the maximum possibility of hospitalization. Given this, improving chronic disease management for post-retirement migrants in eastern China would help to yield a lower hospitalization rate.

These findings shed light on the high rate of hospitalization among older migrants in China. Post-retirement migrants were in need of effective chronic disease management, and a health surveillance system applied to them would be conducive to health promotion and medical resource saving (20).

### Potential reasons for the association and policy implication

The adaptation process regarding daily life, social relationships, and obtaining social support is difficult for older urban migrants (33), who are liable to fall into a state of social isolation and loneliness (34, 35). Migrating in later life is therefore associated with mental health problems, such as depression, anxiety and stress symptoms (32, 36–40). The high association between migration and mental illness may explain why mental illness was excluded in multivariate analyses, as two highly associated variables do not simultaneously exist in regression models. However, previous studies has proved that mental illness indirectly links to physical health impairment and accelerating progression of pre-existing diseases (41), and then cause hospitalization. The mental illness among migrants should be a concern. In order to eliminate social inequalities in mental health outcomes among migrants, more public policies related to mental health services should be established, and community intervention should be prioritized (42).

Patient migration is becoming globally common (43). The eastern region is the most economically developed area in China, and possesses more high-quality medical resources than other regions (24, 44). As a result, patients who could not be diagnosed or cured migrated to eastern China to seek for appropriate treatment. Additionally, eastern China has paced the whole country in providing settlement services for non-local inpatients, hence greatly facilitating hospitalization procedures for migrants. This progress particularly occurs in the Yangtze River Delta region. The combination of regional advantages partly explains why post-retirement migrants in eastern China more inclined to be admitted to hospitals.

However, economic development and improvement of health resources rarely improve access to primary services for Chinese older migrants (15). Migration has significantly reduced migrants' probabilities of hypertension awareness and receiving physician advice (45). The lack of primary care would undoubtedly accelerate the process of health deterioration, consequently leading to hospitalization. The founding that the post-retirement migrants with chronic diseases used more inpatient services is a powerful indicator of potentially insufficient health management, which suggests an efficient system is needed to ensure equal primary care in China.

In view of the above, the authorities in eastern China should develop measures as quickly as possible to guarantee adequate primary health services for migrants. Professional medical examination should be promptly provided to older migrants with unconfirmed chronic conditions. The diagnosed patients should be included in the local system of health management. Moreover, the local-migrant gaps in primary care and outpatient services utilization are normally attributable to the place of insurance participation as well as the categories of health insurance (46). It is essential to perfect the instrument of settlement services for non-local medical treatment and to promote the transfer of migrants' social medical insurance across different regions.

### Limitations

This study has some limitations. First, limited by the content of the questionnaire based on CLHLS, we were able to distinguish the migrants but unable to assess the reasons for migration and the past medical history of this population. The results of regression analysis indicate the content of association as it may not be scientific enough to draw a causal connection by merely clarifying the chronological order. Second, the impact of short-term migration on hospitalization cannot be determined, for merely the older adults who had lived in their current residence for not <2 years were involved in this study. Third, the obtainment of primary care and outpatient services could not be acquired, thus rendering it difficult to directly verify the extent to which the deficiency of these services contributed to the increase in hospitalization. Lastly, the specific causes of hospitalization have not been determined yet, preventing identification of the main diseases affecting the migrant population. A follow-up study is underway to address these limitations.

## Conclusions

Migration is associated with hospitalization among older adults in China. Despite the best socioeconomic status and health habits, the post-retirement urban migrant population is segmented into unique susceptibilities to hospitalization, especially by those with chronic diseases and unconfirmed chronic conditions in eastern China. It is required to supply this vulnerable population with high-quality chronic disease management. Effective policies should be formulated to improve the access of migrants to primary care services, and to narrow the gap in health care services across China. Nevertheless, further studies are necessary to investigate the reasons for migration, and the causal relationships between migration and hospitalization.

## Data availability statement

The original contributions presented in the study are included in the article/supplementary material, further inquiries can be directed to the corresponding author.

## Ethics statement

The studies involving human participants were reviewed and approved by Biomedical Ethics Committee, Peking University. The patients/participants provided their written informed consent to participate in this study.

## Author contributions

HZ conceived and designed the study and wrote the manuscript. YZ supervised the project, critically revised the manuscript for important intellectual content, and procured funding. HZ and NZ analyzed data. JY supervised the project and analyzed the formal. XL reviewed the manuscript. All authors approved the final manuscript.

## Funding

This research was partly supported by the National Key Research and Development Program of China, grant number 2018YFC0114500 and 2018YFC0114506.

## Conflict of interest

The authors declare that the research was conducted in the absence of any commercial or financial relationships that could be construed as a potential conflict of interest.

## Publisher's note

All claims expressed in this article are solely those of the authors and do not necessarily represent those of their affiliated organizations, or those of the publisher, the editors and the reviewers. Any product that may be evaluated in this article, or claim that may be made by its manufacturer, is not guaranteed or endorsed by the publisher.
